# Innovative Air-Preconditioning Method for Accurate Particulate Matter Sensing in Humid Environments

**DOI:** 10.3390/s24175477

**Published:** 2024-08-23

**Authors:** Zdravko Kunić, Leo Mršić, Goran Đambić, Tomislav Ražov

**Affiliations:** 1 Department of Information Systems and Business Analytics, Algebra University, Gradišćanska 24, 10000 Zagreb, Croatia; 2 Rudolfovo—Science and Technology Centre, Podbreznik 15, 8000 Novo Mesto, Slovenia; 3 Department of Software Engineering, Algebra University, Gradišćanska 24, 10000 Zagreb, Croatia; goran.dambic@algebra.hr (G.Đ.); tomislav.razov@algebra.hr (T.R.)

**Keywords:** low-cost particulate matter sensors, outdoor air quality measuring, indicative measuring station, air preconditioning for measuring, preventing sensor readings artifacts, air quality in city

## Abstract

Smart cities rely on a network of sensors to gather real-time data on various environmental factors, including air quality. This paper addresses the challenges of improving the accuracy of low-cost particulate matter sensors (LCPMSs) which can be compromised by environmental conditions, such as high humidity, which is common in many urban areas. Such weather conditions often lead to the overestimation of particle counts due to hygroscopic particle growth, resulting in a potential public concern, although most of the detected particles consist of just water. The paper presents an innovative design for an indicative air-quality measuring station that integrates the particulate matter sensor with a preconditioning subsystem designed to mitigate the impact of humidity. The preconditioning subsystem works by heating the incoming air, effectively reducing the relative humidity and preventing the hygroscopic growth of particles before they reach the sensor. To validate the effectiveness of this approach, parallel measurements were conducted using both preconditioned and non-preconditioned sensors over a period of 19 weeks. The data were analyzed to compare the performance of the sensors in terms of accuracy for PM_1_, PM_2.5_, and PM_10_ particles. The results demonstrated a significant improvement in measurement accuracy for the preconditioned sensor, especially in environments with high relative humidity. When the conditions were too severe and both sensors started measuring incorrect values, the preconditioned sensor-measured values were closer to the actual values. Also, the period of measuring incorrect values was shorter with the preconditioned sensor. The results suggest that the implementation of air preconditioning subsystems in LCPMSs deployed in smart cities can provide a cost-effective solution to overcome humidity-related inaccuracies, thereby improving the overall quality of measured air pollution data.

## 1. Introduction

Intuitively, clean air is necessary for good health. Air pollution happens from both natural (for example, volcanoes and decaying organic mass in oceans) and man-made (for example, burning wood for heating, fuel-based engines, and industrial byproducts) sources. Throughout the decades, medical researchers have been studying the impact of polluted air on human health. Air pollution has been linked to up to 9 million premature deaths globally, in 2015 [1]. In 2015, the World Health Organization adopted a landmark resolution on air quality and health. In that resolution, air pollution was identified as a risk factor for noncommunicable diseases such as stroke, asthma, lung cancer, etc., putting it on the same level as other major health risk factors such as unhealthy diets and tobacco smoking [2]. In 2021, the WHO published their latest guidelines [3]. Based on the WHO resolutions and guidelines, the European Union has adopted its own air quality standards in the form of the Ambient Air Quality Directive (AAQD) 2008/50/EC to set air quality standards for 12 air pollutants: sulfur dioxide, nitrogen dioxide / nitrogen oxides, particulate matter (PM_10_, PM_2.5_), ozone, benzene, lead, carbon monoxide, arsenic, cadmium, nickel, and benzo(a)pyrene [4]. In 2020, the EU updated the 2008/50/EC to resemble the new WHO guidelines more closely [5].

When it comes to measuring air quality, the AAQD documentation recognizes fixed measurements and indicative measurements, followed by possible data modeling or postprocessing with or without the use of machine learning methods [6]. Fixed measurements are taken by the fixed measurement stations, rather expensive, complex, and precise pieces of equipment placed in big, permanent installations. These fixed stations are also known under the names of conventional, regulatory, or reference measuring stations. Although they provide reliable and accurate data, due to their size and price, it is not possible to have them placed in every critical zone, as required by [6]. Also, these stations provide hourly measures. Hourly measurements are inadequate for early detection scenarios, as the infrequency of updates can delay responses to emerging threats, whereas higher-frequency measurements, such as those taken every minute, would significantly enhance the ability to identify and react promptly. In contrast, indicative measurements are taken by indicative-measurement stations, relatively cheap and less accurate devices in comparison to fixed stations. The basis for indicative stations is low-cost particulate matter sensors, often abbreviated as LCPMSs, so these stations are often called LCPMS measuring stations. The goal of the AAQD is that fixed measurements should be supplemented by indicative measurements (and possible modeling and/or objective estimation) to enable point data to be interpreted in terms of geographical distribution of concentrations and the reduction of the required number of fixed stations, resulting in better data, lower cost, and real-time monitoring [6].

It is well established that indicative stations suffer from certain drawbacks, mostly in terms of component quality, measurement reliability, and their relative lifetime [7]. There are many efforts to overcome these problems without raising the price of the indicative device, branching in different directions, and trying to solve different issues [7,8]. This paper is focused on overcoming one such shortcoming of indicative stations.

When it comes to measuring particulate matter concentrations, specifically PM_2.5_ and PM_10_, there are multiple measuring techniques. Out of all the available techniques, all the LPCMS employ a light-scattering method, due to its low cost [9]. The basic idea is simple: when a particle passes through a light beam, part of the light deviates from the original path, producing a scattering [9]. The characteristic intensity of scattered light is precisely predicted by the Mie theory, and by analyzing it, the particle size distribution can be determined [9]. It was reported that indicative stations employing a light scattering-based particulate matter sensor can exhibit good performance with high accuracy, if they have been calibrated in the laboratory by the manufacturer and in-field by the implementor [9].

Even with calibrations, it was reported that all LCPMSs report serious degradation in accuracy in high relative-humidity conditions, causing the device to significantly overestimate particle counts [9,10,11,12]. Simply put, high relative humidity causes certain aerosol particles present in the air to consume water vapor from the air. This happens because of the hygroscopicity property of some particles, that is, their ability to absorb water (for example, it comes as no surprise that NaCl particles in the aerosol will absorb water) [10]. This process causes smaller particles to grow because of the absorbed water, therefore scattering the light differently and causing the sensor to incorrectly put them in the larger bin than they really should be in. Won et al. explain this hygroscopic growth represented as the particle size hygroscopic growth factor [13]:

(1) $$GF\left(RH\right)=\frac{D\left(RH\right)}{D_{d}}$$

Equation (1) shows that the hygroscopic growth factor (*GF*) at the given relative humidity (*RH*) is defined as a ratio of the diameter of the particle (*D*) at that same relative humidity to that of the particle (*D_d_*).

As a result of this hygroscopic growth, the light-scattering sensors overestimate the PM_2.5_ and PM_10_ concentrations, causing possible health hazard alerts, although most of those hygroscopic particles are mostly condensed water and do not have any adverse effects on health [9]. To remove these hygroscopic effects, fixed stations are equipped with huge presampling chambers (dryers) that bring the air to a specific temperature and to relative humidity values before letting it reach the sensors [10]. LCPMSs cannot afford this approach, due both to size and to price constraints (dryers are big and cost a lot), so they most often deal with this issue in a different way. Some LCPMSs do the following:

- Employ different algorithms to compensate elevated measured levels (including averaging of values or machine learning models [14]);
- Detect such environmental conditions and report that no reliable measurements are available for that period [15];
- Use calibration methods based on the relative humidity values and K-value parameter for hygroscopicity of the air mixture [16].

This paper takes the approach of using low-cost dryers to remove the water from the air and reduce the negative effects of the relative humidity on the reported results. The aim is to improve the accuracy of low-cost indicative stations in measuring particulate matter, while keeping the equipment cost low and in the acceptable range for the low-cost indicative devices. It does so by presenting a novel design of the inlet that uses strategically placed heaters to preheat the air before it reaches the sensor. More specifically, this paper puts forward two hypotheses:

- H1: it is possible to predict when the hygroscopic particles will be formed based on the relative humidity and temperature values;
- H2: a proposed novel inlet use will reduce the effect of the hygroscopic particles on the measured PM_2.5_ and PM_10_ levels, giving more accurate measured values.

Section 2 presents related work on the effect of hygroscopic particles on measured levels of PM_2.5_ and PM_10_. Section 3 describes the indicative station containing the novel inlet and particulate matter sensor. It also describes the testbed for the system and testing scenarios. Section 4 presents the findings and draws conclusions on the approach taken. Finally, a conclusion is given.

## 2. Related Work

Conventional, fixed, regulatory, or reference measuring stations provide accurate measurements of air pollutants, but they are physically big, require heavy investment, and qualified personnel to run, making it hard to deploy them on a large scale. To better assess local pollution levels and obtain almost instantaneous readings, an addition to conventional stations is proposed; the low-cost sensor-based, or indicative measuring stations are envisioned to complement fixed stations. They are cheap, small-sized, easily installed, offer real-time coverage, and can easily be deployed on a dense scale, although they do not necessarily provide the same measurement reliability or lifetime. At the heart of these indicative stations are the solid-state chemical sensors for gaseous air-pollution monitoring and the scattering-based particulate matter sensors for detecting mainly PM_2.5_ and PM_10_ [9], with this paper focusing on the latter. There are many efforts in the scientific community and industry to improve these indicative measuring stations to achieve more reliable and accurate measurement of particulate matter levels over longer periods. One direction of research is designing, building, and investigating low-cost particulate matter sensors (LCPMSs). As stated by Alfano et al., the number of different peer-reviewed LCPMS models went from 7 in 2017 up to 50 in 2020, showing a huge increase in available options [9]. Raysoni et al. give a review of the literature on the usage of low-cost sensors to measure particulate matter [17].

All LCPMSs are pre-calibrated by the manufacturer by using laboratory-level calibration aerosols [18]. In these calibrations, the environment is carefully controlled, most notably the temperature, relative humidity, and aerosol shape and concentration. According to [9,14], three tested sensors (Samyoung DSM501A, Shinyei PPD42NS, and Sharp GP2Y1010AU0F) exhibited high linearity against the reference instrument, with *R*^2^ up to 0.9755, but the test also showed that sensor readings were affected by the humidity and particle size and composition. Vajs et al. have shown that the tested LCPMSs (Sharp GP and Sharp DN) showed a correlation *R*^2^ of above 0.97 [19]. In a testing chamber capable of simulating environments with temperatures from 5 °C to 40 °C, relative humidity from 10% to 90%, and stable pollutant concentrations, authors show that an LCPM sensor (HPMA115S0 Honeywell) exhibited *R^2^* values above 0.96 [9,20,21].

However, although in laboratory environments LCPMSs display high correlation with reference instruments, it was shown that aerosols used in the testing chambers differ significantly from aerosols found in outdoor environments [18,22]. The studies [9,22] show that laboratory-calibrated-only devices can commonly exhibit *R*^2^ values lower than 0.5 in real outdoor environments, so field-calibration is required. Additionally, to improve LCPMS performance, postprocessing calibration strategies are implemented and they usually include models with corrections for hygroscopicity, linear regression models, and other machine learning algorithms [9,23,24,25,26,27].

Many authors have reported interference caused by relative humidity. Wang et al. report that the water in the air affected the accuracy of the sensors in three ways, most notably by absorbing light and causing an overestimate of particle mass concentrations due to the reduced light intensity received by the phototransistor [14]. They also reported that the temperature did not play any significant role. Some authors show that high relative humidity can cause serious disruption to sensors’ measurements [15,28,29].

The most studied approach in literature for mitigating the influence of relative humidity is that of applying different correction algorithms. Liu et al. propose a correction factor dependent on relative humidity to be applied to measured data to remove the effects of high relative humidity [30]. Di Antonio et al. introduces a correction algorithm where relative humidity effects can be better described by taking into account the detailed particle size profile, allowing the algorithm to account for the high relative-humidity artefacts on the measured concentration levels [11]. Vajs et al. apply different machine learning methods to improve the calibration algorithms to increase the accuracy and mitigate the impact of relative humidity on the readings [19].

## 3. Materials and Methods

### 3.1. Design of the Novel Indicative Air-Quality Measuring Station

In this paper we present a novel indicative air-quality measuring station design. The station contains sensors to measure both gases (like carbon monoxide, etc.) and particulate matter, including substances like benzene and lead. For this paper, only the part of the station relevant to measuring particulate matter, specifically PM_2.5_ and PM_10_, will be discussed.

While the overall design of the station incorporates various elements to ensure accurate readings, the heating system within the conditioning tunnel plays a pivotal role in addressing the challenges posed by high relative humidity. This section explains deeper technical aspects of the heating system, exploring its design rationale, control mechanisms, and integration with other components of the station:

- Heater Design Considerations

The spiral heater was chosen for its optimal heat-distribution characteristics and energy efficiency. The spiral configuration maximizes the surface area in contact with the airflow, ensuring uniform heating of the sample air. This design also allows for rapid temperature changes, enabling the system to respond quickly to fluctuations in ambient conditions or airflow rates.

- Advanced Temperature-Control Algorithm

The proportional–integral–derivative controller (PID) controller governing the heater incorporates adaptive tuning algorithms to optimize performance across varying environmental conditions. This advanced control system not only maintains the target temperature, but also minimizes overshoot and settling time, ensuring stable heating even during rapid changes in ambient temperature or humidity. The controller’s parameters are automatically adjusted based on historical performance data, improving the system’s responsiveness over time.

- Power Management and Efficiency

To optimize energy consumption, the heating system includes a power management module. This module modulates the heater’s power input based on real-time requirements, reducing energy usage during periods of low humidity or when ambient temperatures are closer to the target. This feature enhances the overall efficiency of the station, making it more suitable for long-term, unattended operation in various smart-city deployments.

- Maintenance Considerations

The heating system is designed with ease of maintenance in mind. The modular design of the heating element allows for easy replacement or upgrades without necessitating a complete overhaul of the station, contributing to the system’s long-term reliability and adaptability.

**Figure 1:**
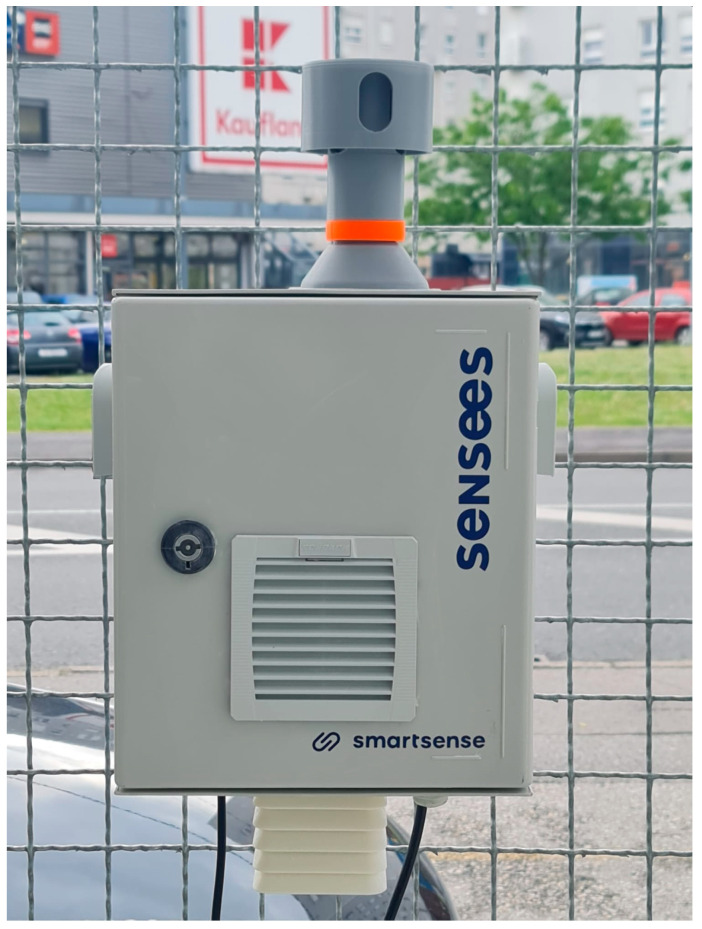
Novel LCPMS-based indicative station.

The heart of the station is the low-cost particulate matter sensor Alphasense OPC-N3 Optical Particle Counter, based on the light-scattering technique. The performance of this sensor has been extensively studied [14,31] and, as with other LCPMSs, it has been shown that it suffers from the effects of high relative humidity. Therefore, a novel design that includes air heating was developed for the measuring station. Figure 1 shows the full station, designed and manufactured to reduce the effects of the relative humidity on particulate matter measurements. 

**Figure 2:**
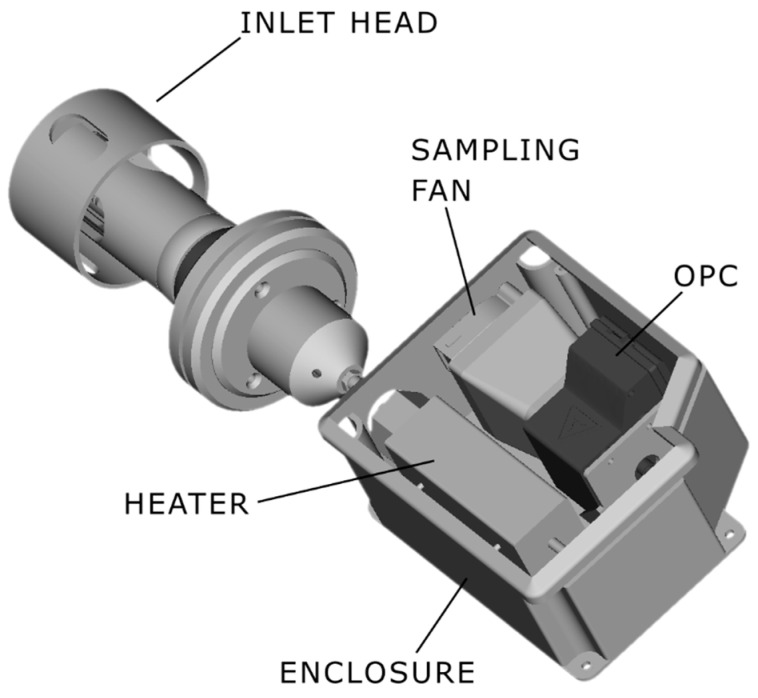
Main parts of the particle-matter measurement subsystem.

For this paper, the top-most part of the station is interesting, because it houses the inlet of the particle measurement subsystem. Figure 2 shows the main parts of that subsystem.

**Figure 3:**
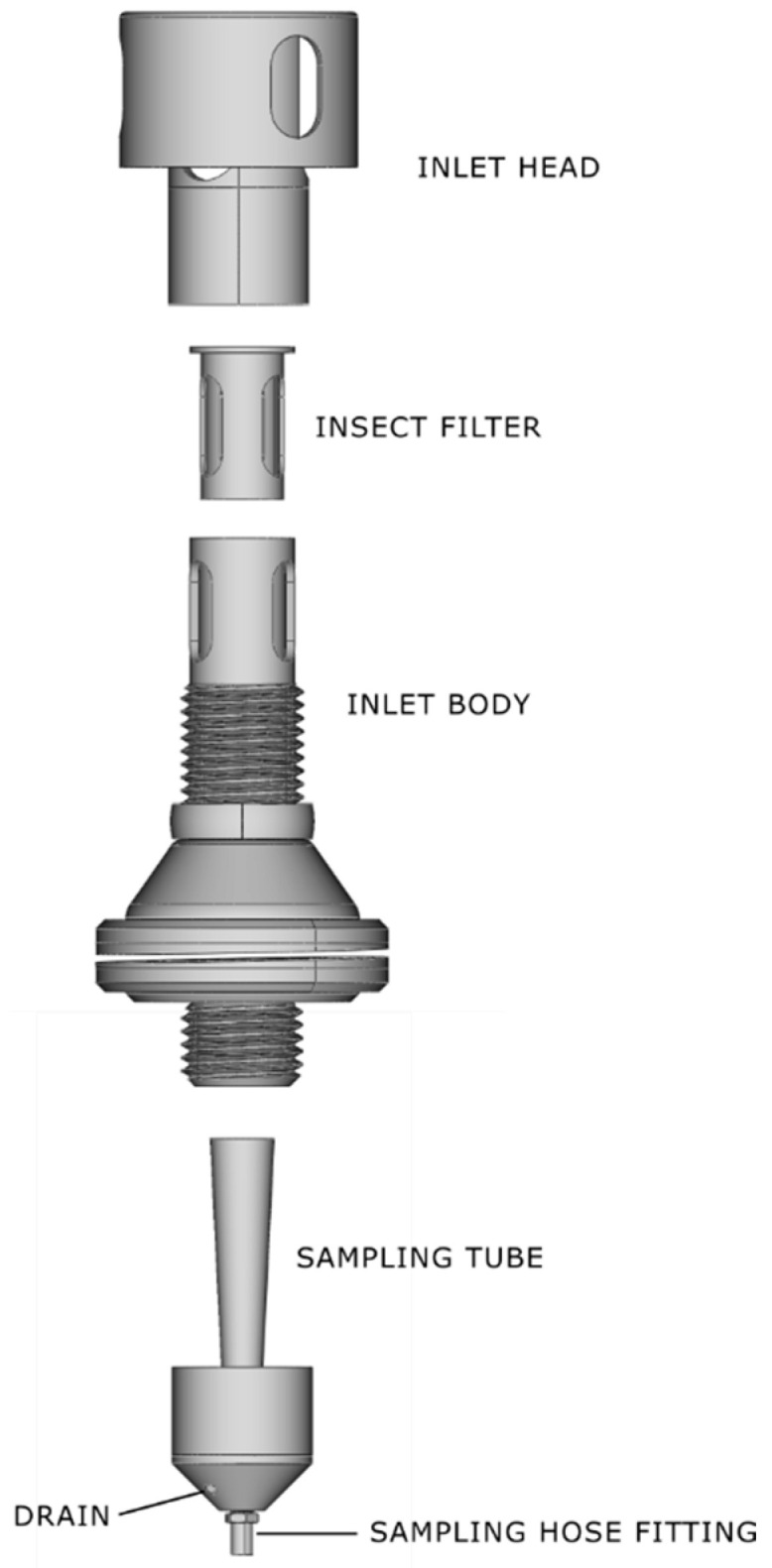
Detailed view of the particle-matter measurement subsystem.

The air enters the inlet head, then goes through the conditioning tunnel, and enters the measuring chamber, where the Alphasense OPC-N3 sensor is located. This design forces the air to pass through a conditioning tunnel with the goal of bringing microclimate parameters of the air, such as temperature and relative humidity, to a preferred range, considering the sensor technology used. Figure 3 shows a more detailed view of the particle-matter measuring subsystem.

**Figure 4:**
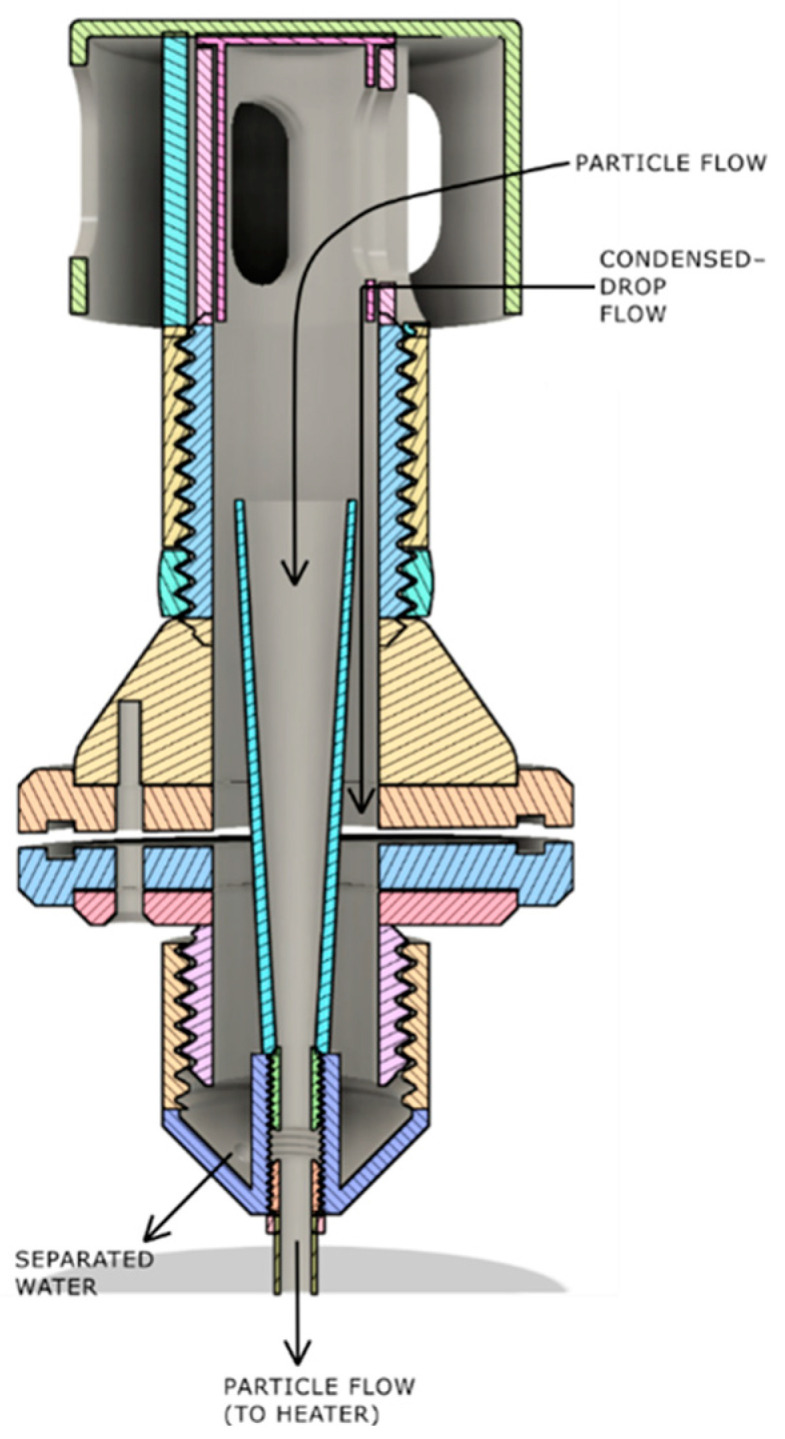
Subsystem for removing condensed water from the intake path.

The air enters the subsystem through a sampling head, which is designed to enable easy entry of PM_10_ and lower sized particles, while preventing coarser grained dust and sand from entering the sampler. At the same time, it provides protection from the rain and sun. Inside the head there is a filtering net to prevent the entry of insects, as well as other macro particles like pollen. Particular care is taken while designing the inner sampling tube. It maximizes the air flow from the sampling head, while allowing condensed water to drain over the sides of the main inlet body down to the drain hole. From the drain hole, water is drained out using the drain tube, through the monitoring station, all the way to the dedicated opening at the bottom. This is especially important for preventing condensed water droplets from entering the optical-particle-counter sampling path, because the heating system cannot remove big, condensed drops of water from the intake path. Figure 4 shows this in a graphical way.

From the sampling head, the air goes to the conditioning tunnel. The main component of the conditioning tunnel is a spiral 30 W heater. As the air passes through, the heater raises its temperature. When the air reaches the particle counter, it is heated, and most hygroscopic particles have been significantly reduced in size. That causes similar effects to that in big preconditioning dryers in reference stations, and allows the particle counter to measure more accurate values, while keeping the size, price, and complexity of the station low. In the context of measuring particle concentration, there is a pertinent question as to whether volatile particles can evaporate, leading to an underestimation of particulate matter concentrations. The evaporation of volatile and semi-volatile components is a well-documented phenomenon that can significantly impact the accuracy of particulate matter measurements.

Sample-flow control turned out to be an important aspect of the heated sampling system, so both the heater and the air flow through the system had to be regulated. The main problem was that adding any additional tubing, heaters and ducts to the OPC N3 causes its sample flow to drop from a nominal 4–6 $\frac{mL}{s}$ to 0.7–0.8 $\frac{mL}{s}$. Because the flow is used in OPC internal algorithms to calculate mass of particles per volume, the low flow results in a greater scattering of the measurements, depending on the particle concentration. The problem was solved by adding an external sampling fan, as shown in Figure 2. This fan can generate significantly higher air flow than the one in OPC N3, thus allowing the system to control the sample flow at the intended 4–6 $\frac{mL}{s}$, by using the PID controller manufactured by Smart Sense (Zagreb, Croatia). In general, the flow is affected by wind, air pressure, temperature and other factors, so by being able to stabilize the flow and control the heater temperature, the system was able to achieve significant improvements in measurement precision and reliability. The heater control was set to keep the temperature below 45 °C and the sample flow rate at 5 $\frac{mL}{s}$.

### 3.2. Rationale for Heating the Air

The rationale for the raising of the temperature is explained next. Ref. [32] states that the relative humidity (*RH*) shows how much water is in the air compared to the maximum water vapor the air can hold at a specific temperature, expressed as a percentage. A dew point is defined as the temperature of the air required to achieve *RH* = 100%. Below this temperature, the air cannot hold any more water so the water will start to condense. Equation (2) [32] shows the relation between dew point, air temperature, and relative humidity:

(2) $$RH=\frac{e^{\frac{\beta D_{p}}{\lambda +D_{p}}}}{e^{\frac{\beta T}{\lambda +T}}}\;*\;100$$

**Figure 5:**
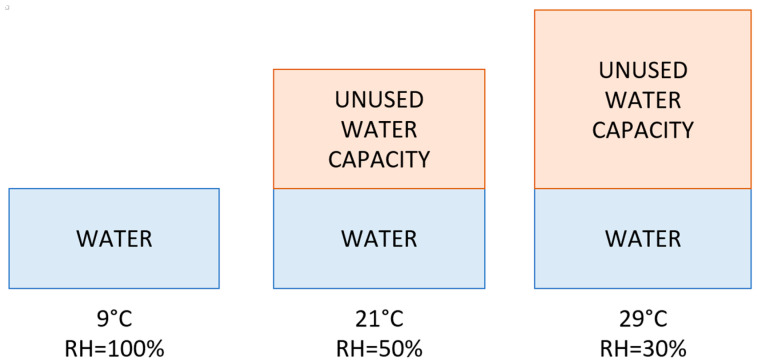
Relationship between relative humidity and temperature.

In Equation (2), *D_p_* is the dew-point temperature, *T* is the air temperature, and *β* = 17.625 and *λ* = 243.04 °C are revised Magnus coefficients recommended in [32]. The said equation suggests that the increase in the temperature will decrease the relative humidity. That concept is shown visually in Figure 5.

Figure 5 graphically shows that, although the amount of water in the air remains the same, the increase in temperature allows more water to be present in the air and therefore lowers the relative humidity.

Progressing from that, the authors in [33] show that lowering the relative humidity will cause hygroscopic particles to decrease in size, which is exactly the purpose of the design.

### 3.3. Experiment Setup

**Figure 6:**
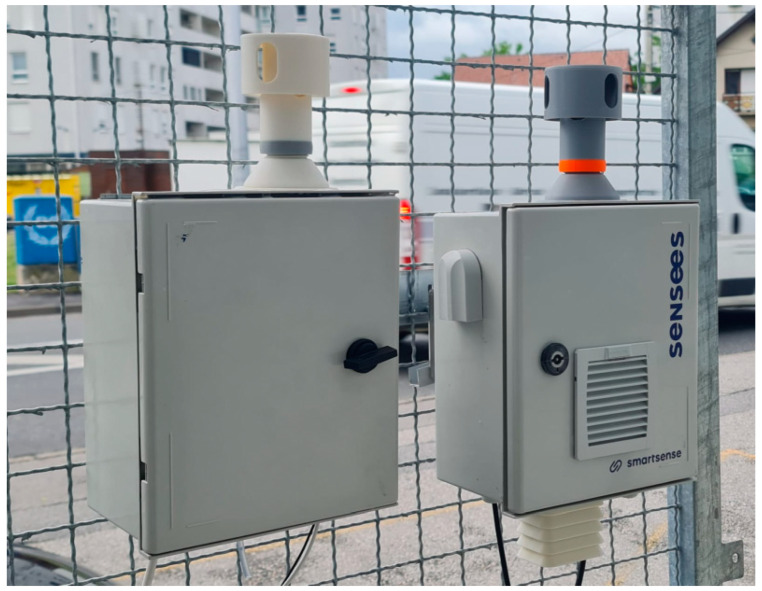
Experiment setup.

The experimental setup included two monitoring stations with exactly the same configuration, except that one of them was equipped with the subsystem for heating the air proposed in this paper, and the other one was not. Otherwise, they shared the same mechanical and electronic components and ran the same software. Figure 6 shows the placements of both stations, the one equipped with the subsystem for heating the air proposed in this paper being on the right.

The monitoring stations were equipped with Alphasense sensors, five electrochemical gas sensors (NO, NO_2_, CO, O_3_ and SO_2_), and one OPC N3 particle counter measuring the PM_10_, PM_2.5_ and PM_1_ types of particles. Additionally, each monitoring station included external meteorological sensors (temperature, relative humidity, and atmospheric pressure) enclosed in a radiation shield. To keep both stations as similar as possible, one monitoring station was equipped with the new heated sampling inlet, and the other was equipped with the same inlet, just without the heating.

Both stations were placed in the same installation location, making sure that their PM sampling inlets were as close as possible and at the same distance from the ground. In this experiment, data from the reference monitoring station was not used, although the GRIMM EDM 264 automatic sampler instrument for monitoring and supervision of the experiment was used. The reason for this was to show the difference in performance of the otherwise identical optical particle counters with regards to conditioning of the sampler. In other words, this experiment was not interested in the absolute accuracy of the data, but rather the relative comparison between the two.

The installation location and the season were selected to provide regular periods, with high relative humidity combined with that of normal conditions. One important goal of the experiment was not only to prove the benefits of the heating of the input samples when the conditions require it, but also to make sure there are no side effects of this setup in normal conditions when the heating is not required. The results were an important input for the design of the heating regulation-control firmware. The main question was whether to have strict humidity and temperature thresholds, including hysteresis, for switching the heater on and off, or whether it were perhaps a better strategy to have the heater on all the time, with the set temperature target, thus avoiding negative effects of sudden changes in temperature and humidity on the measurements.

The testing was performed in the winter and spring seasons, allowing the monitoring stations to be exposed to many different conditions, ranging from the cold weather in winter to the periods of huge daily temperature and humidity swings during the spring season. Also, an urban location was chosen, next to a busy street, which provided good daily dynamics of the particle concentrations, so that the devices were exposed regularly both to the low (clean air) and elevated levels of the PM concentration. At no point during the test period did the particle concentration at the test location reach high levels that could pose health issues. This was also confirmed by the GRIMM EDM 264 instrument, manufactured by Durag Group (Hamburg, Germany).

Both monitoring stations also contain the power supply subsystem and the gateway. The gateway provides 4G connectivity and has the task of configuring and reading the OPC N3 counter. The particles are sampled every couple of seconds, and then one-minute averages are calculated. One-minute averages are then sent to the backend server, where they are stored and can be visualized on the frontend application. The conducted experiments have shown that the minute intervals are sufficient, providing enough data granularity on one side, while filtering out the volatile swings of PM concentration characteristic of suspended particles.

## 4. Results

The experiment was conducted over a period of 19 weeks, with two weather stations included—one conditioned and one unconditioned. The sensors measured the concentrations of PM_1_, PM_2.5_, and PM_10_ particles at a sampling rate of 1 min, and the difference between the two sensors was calculated.

**Figure 7:**
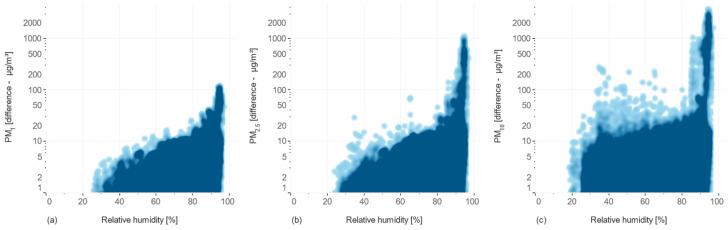
The incidence relationships of the relative humidity and the difference between unconditioned/conditioned sensors for measuring concentrations of (**a**) PM_1_, (**b**) PM_2.5_, and (**c**) PM_10,_ respectively.

The heatmaps in Figure 7 show the relationship between the difference in measurements between the unconditioned and conditioned sensors, and the relative humidity. The results indicate that larger particles (PM_10_) are more sensitive to increased humidity in the outside air.

For PM_1_ and PM_2.5_, the difference between the unconditioned and conditioned measurements starts to increase gradually from around 25% relative humidity. This gradual increase suggests that hygroscopic growth begins to affect these smaller particles at lower humidity levels. As relative humidity increases, water vapor begins to condense on these particles, increasing their size and causing the unconditioned sensors to overestimate particle concentration. The preconditioning subsystem effectively mitigates this effect, hence the observed difference. For PM_10_, the difference becomes noticeable even at around 30% relative humidity, and shows a more abrupt increase compared to PM_1_ and PM_2.5_. This could be because larger particles, such as those in the PM_10_ range, might already contain more hygroscopic materials, which respond more quickly to changes in humidity. Additionally, PM_10_ particles might include larger droplets or aggregates of smaller particles, which can exhibit more pronounced hygroscopic growth even at lower humidity levels.

Across all particle sizes (PM_1_, PM_2.5_, and PM_10_), there is a marked increase in the difference at very high humidity levels, especially near 100%. This sharp rise is indicative of extreme hygroscopic growth, where particles absorb significant amounts of water, leading to substantial overestimation of particle concentration by unconditioned sensors. The preconditioning subsystem helps reduce these effects by lowering the relative humidity of the air before it reaches the sensor, thus minimizing measurement errors.

**Figure 8:**
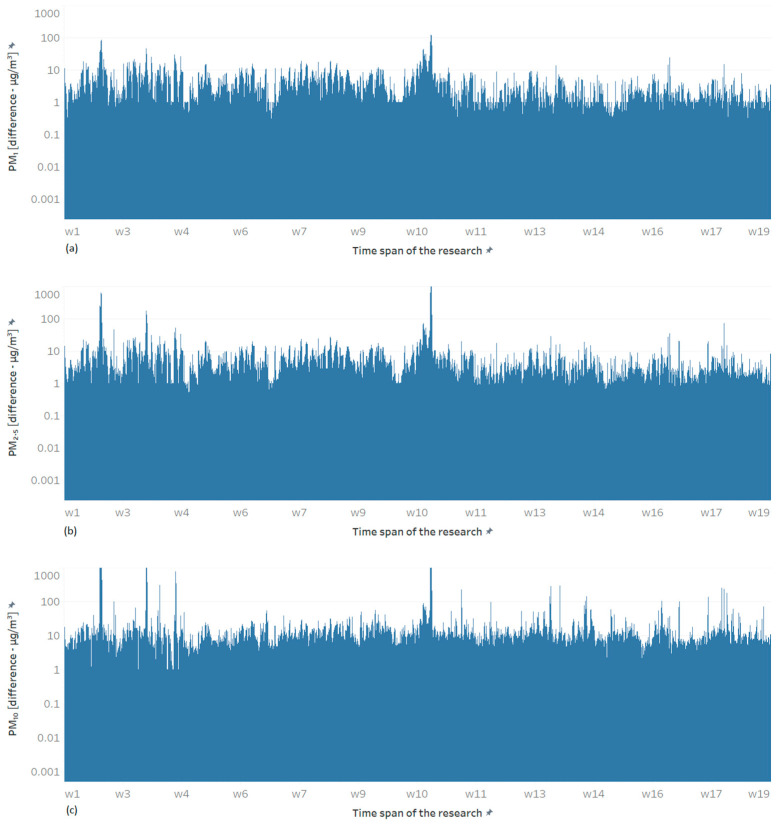
The absolute differences in the sampled values between the conditioned and unconditioned sensors over the entire experimental period for (**a**) PM_1_, (**b**) PM_2.5_, and (**c**) PM_10,_ respectively.

To further analyze the difference between the conditioned and unconditioned sensors, the absolute differences in the sampled values were visualized over the entire experimental period for each particle size (Figure 8). The results confirm that the differences are larger for larger particles, with PM_1_ concentration differences ranging up to approximately 100 particles, PM_2.5_ ranging up to 1000, and PM_10_ often close to or over 1000.

**Figure 9:**
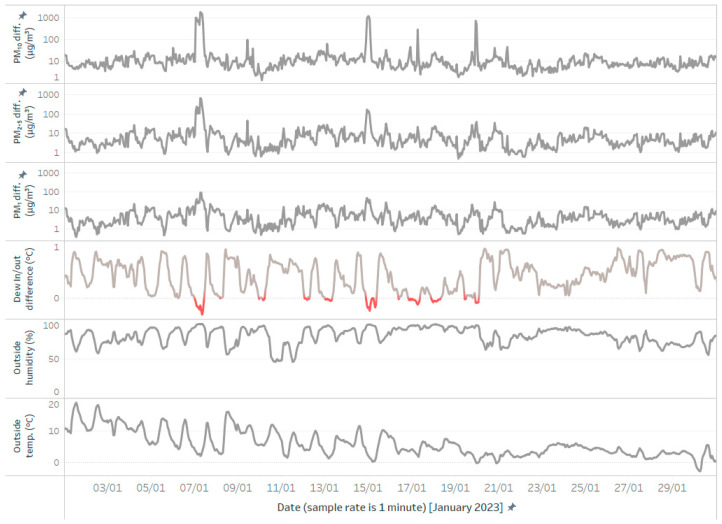
Comparative overview of conditioned/unconditioned sensor-reading differences, dew point outside/inside difference, outside humidity and outside temperature, in January 2023.

The prominent peaks observed in Figure 8 were further analyzed in the context of dew point, the difference between measurements of unconditioned and conditioned sensors, outside humidity, and outside temperature (Figure 9). The results show that the highest measurement error is observed for the largest particles (PM_10_), and this error is correlated with negative values of the dew-point difference (i.e., when the outside dew point is lower than the inside dew point). 

**Figure 10:**
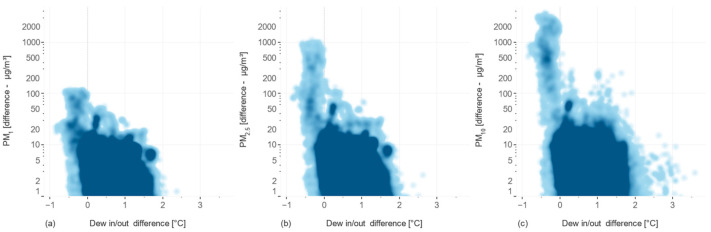
The incidence relationships of the dew-point difference between inside/outside air and the difference between unconditioned/conditioned sensors for measuring concentrations of (**a**) PM_1_, (**b**) PM_2.5_, and (**c**) PM_10_, respectively.

The relationship between the dew point difference and the difference between unconditioned and conditioned sensor measurements is further illustrated in Figure 10. The results show a clear correlation between the dew-point difference and the sensor differences, with larger particles (PM_10_) being more affected by changes in dew point.

Congruence between measurements conducted with conditioned and unconditioned sensors can be classified into four classes: (a) both sensors show relatively stable, comparable PM concentrations; (b) the conditioned sensor is stable, but the unconditioned is not; (c) both sensors are unstable, but the unconditioned sensor is much worse; and (d) both sensors are unstable, but the conditioned sensor’s instability duration is much shorter. The following figures show examples for each category. 

**Figure 11:**
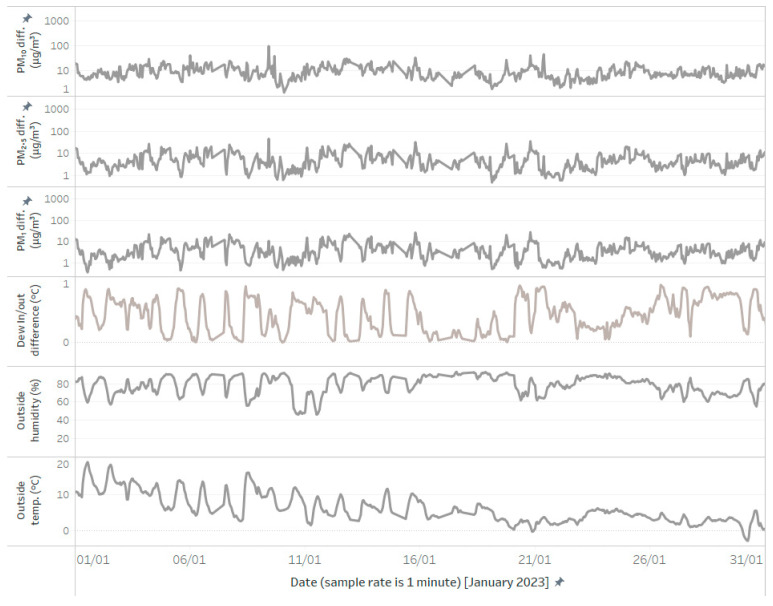
Both sensors are stable, regardless of high humidity, due to a dew point higher than the actual temperature. This visualization is similar to Figure 9, but samples with negative values of dew in/out difference are filtered out.

The first identified class is characterized by a positive difference in dew points, where the outside-air dew point is higher than the inside-air dew point. In these situations, even when the relative humidity is high, both the conditioned and unconditioned sensors can measure comparable values. This indicates that when the outside air temperature is higher than the conditioned air temperature, and the dew point is not low, the sensors can provide stable and reliable measurements, despite the high relative humidity, as shown in Figure 11.

**Figure 12:**
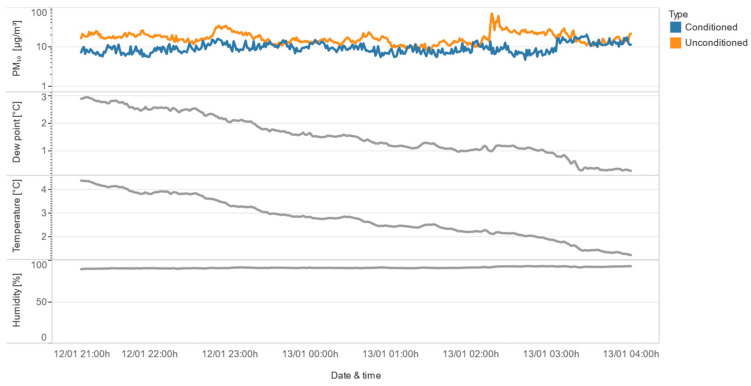

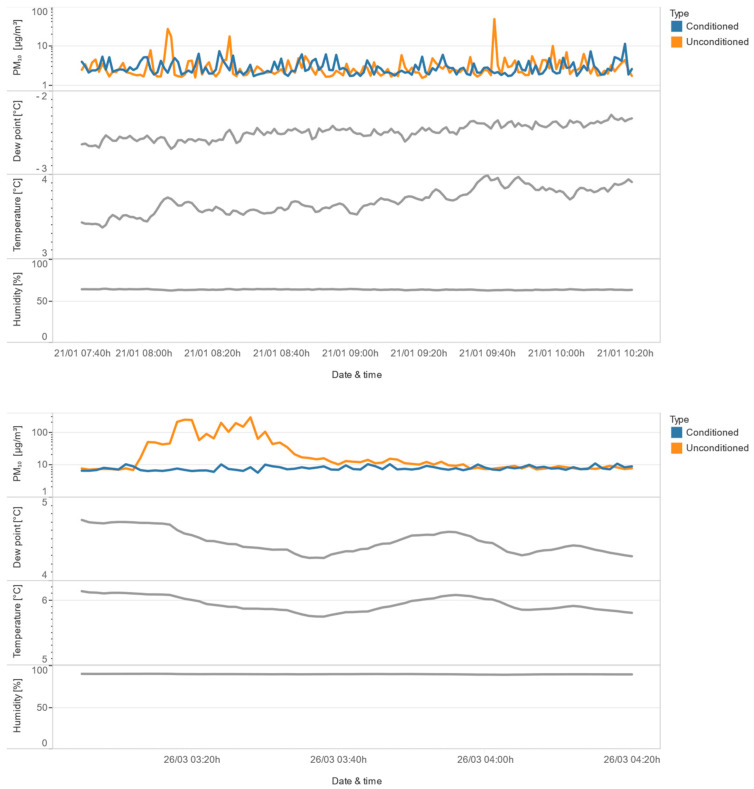
Detailed examples of situations in which the conditioned sensor measures stable values, while the unconditioned sensor is unstable.

In the second class of congruence, the conditioned sensor maintains stable measurements, while the unconditioned sensor exhibits instability, as illustrated in Figure 12. This suggests that the conditioning of the air effectively mitigates the influence of high relative humidity, allowing the conditioned sensor to provide reliable data even when the unconditioned sensor is affected by the environmental conditions.

**Figure 13:**
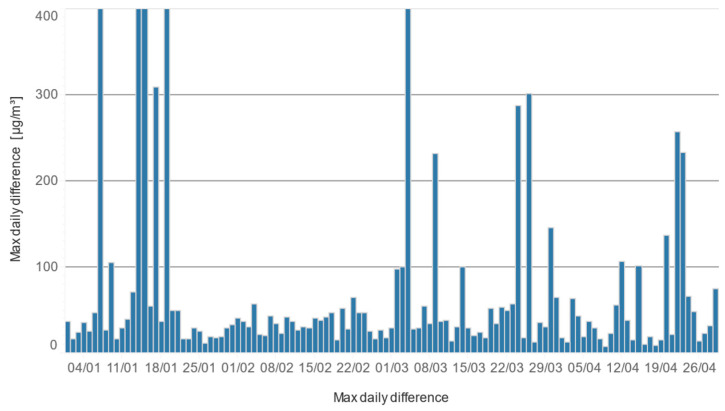
PM_10_ concentration difference between conditioned and unconditioned sensor during complete measurement period on the daily level. The y-axis scale is limited to 400 µg/m^3^ for better readability.

During the 19-week period, there were seventeen days during which the concentration difference between the conditioned and unconditioned sensor exceeded 100 µg/m^3^: six days in the range 101–200 µg/m^3^, four days in the range 201–300 µg/m^3^, two days in the range 301–400 µg/m^3^, and five days over 400 µg/m^3^, as shown on Figure 13.

**Figure 14:**
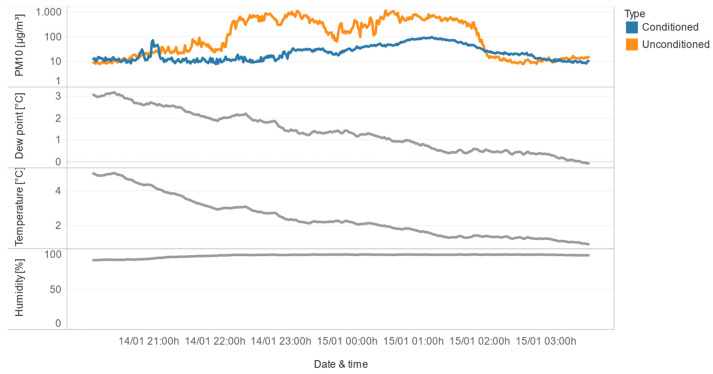
Both sensors are unstable, but the conditioned sensor is better.

The third class represents a more extreme scenario, where both the conditioned and unconditioned sensors show instability. However, as shown in Figure 14, the conditioned sensor exhibits a much smaller measurement instability compared to the unconditioned sensor. This indicates that the conditioning of the air helps to reduce the impact of high relative humidity, even when both sensors are affected to some degree.

**Figure 15:**
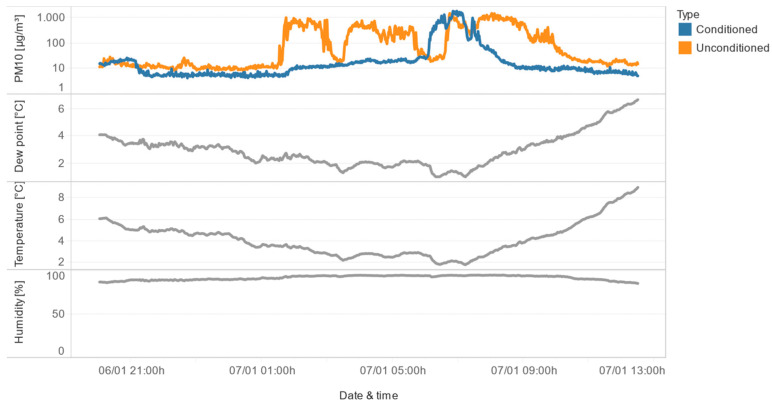
Both sensors are unstable, but the conditioned sensor is unstable for a shorter period.

The final class of congruence includes situations where both sensors show instability, but the duration of the conditioned sensor’s instability is much shorter than the unconditioned sensor’s. As illustrated with an example in Figure 15, the conditioned sensor is unstable after a sudden drop in the dew point near 0 °C, while the unconditioned sensor remains unstable for a longer period. This suggests that the conditioning of the air helps to mitigate the effects of high relative humidity, particularly during rapid changes in environmental conditions.

These four classes of congruence demonstrate the effectiveness of the conditioned sensor in improving the reliability and accuracy of particulate matter measurements, especially for larger particles that are more sensitive to changes in relative humidity. The results show that the conditioning of the air can help mitigate the negative effects of high relative humidity on sensor performance, allowing for more stable and accurate measurements across a range of environmental conditions. 

Furthermore, the findings suggest that the conditioned sensor is particularly beneficial in situations where the outside air temperature is higher than the conditioned air temperature, and the dew point is not low. In these cases, the conditioned sensor is able to provide stable and reliable measurements, even when the unconditioned sensor is affected by the high relative humidity. 

**Figure 16:**
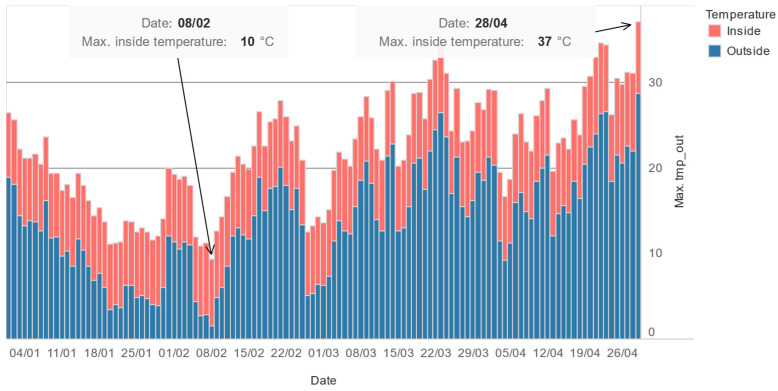
Maximum daily outside (input of the tube) and inside (sensor chamber) temperatures.

The inside- and outside-temperature ranges throughout the whole experiment are shown on Figure 16.

The ability of the conditioned sensor to maintain stability and accuracy during periods of high relative humidity is a significant advantage, as it allows for more reliable monitoring of particulate matter levels in real-world environments. This is especially important for applications where accurate and consistent data are crucial, such as air quality monitoring and environmental research. 

Additionally, the insights gained from the different congruence classes can inform the development of more advanced data processing and correction algorithms, which can further enhance the performance of low-cost particulate matter sensors. By understanding the specific conditions under which sensor instability occurs, researchers and engineers can work to develop more robust and adaptive sensor systems that can reliably operate in a variety of environmental conditions. 

Rainy conditions inherently lead to elevated humidity levels, often nearing 100% relative humidity. This environment poses a significant challenge for particulate matter sensors due to the hygroscopic growth of particles, which is directly influenced by high humidity. Our study specifically addresses this challenge through the implementation of a preconditioning subsystem that effectively reduces the relative humidity of the incoming air before it reaches the sensor.

Since rain represents an extreme manifestation of high humidity, the scenarios we tested encompass the conditions present during rainfall. The results obtained demonstrate that our preconditioning approach successfully mitigates the impact of high humidity, including the conditions observed during rainy periods. As such, the performance of the system during rain is adequately captured within the broader context of our high-humidity testing, providing a comprehensive understanding of its effectiveness across a range of environmental conditions. Overall, the results of this study demonstrate the potential for using conditioned-air inlets to improve the accuracy and reliability of low-cost particulate matter sensors, paving the way for more widespread and effective air-quality monitoring solutions. 

Incorporating preconditioning subsystems into LCPMSs deployed in smart cities could greatly improve the quality of air pollution data, enabling more accurate monitoring and better informed decision-making. This advancement not only supports the development of more effective environmental policies, but also contributes to the overall resilience and sustainability of smart cities.

## 5. Conclusions and Future Work

This paper demonstrates the effectiveness of using air conditioning to improve the accuracy and reliability of low-cost particulate matter sensors (LCPMSs) under different environmental conditions, particularly high relative humidity. The proposed novel inlet design, which incorporates strategically placed heaters to heat up the air, and drains to remove the condensed water, significantly mitigates the hygroscopic effects on particulate matter measurements, leading to more accurate readings of PM_2.5_ and PM_10_ levels. By heating the incoming air and reducing relative humidity before it reaches the sensor, the preconditioning subsystem significantly improves the reliability and stability of PM measurements, especially for larger particles (PM_10_), which are more sensitive to humidity changes.

A statistical analysis of the 19-week measurement period reveals that the preconditioned sensor outperformed the non-preconditioned sensor in terms of measurement accuracy and stability. During this period, there were 17 days where the concentration difference between the two sensors exceeded 100 µg/m³, with the majority (11 days) showing differences greater than 200 µg/m³. In these cases, the preconditioned sensor consistently measured values closer to the actual PM concentrations, with a shorter duration of instability compared to the non-preconditioned sensor. 

The minimum inside temperature achieved during the whole 19-week period of measurements was 9.8 °C, and the maximum was 36.7 °C.

The overall results can be categorized into four classes, based on the congruence between the preconditioned- and non-preconditioned-sensor measurements:

- Both sensors show stable, comparable PM concentrations when the outside-air dew point is higher than the conditioned-air dew point and the relative humidity is not excessively high.
- The preconditioned sensor maintains stability, while the non-preconditioned sensor exhibits instability, demonstrating the effectiveness of air preconditioning in mitigating the influence of high humidity.
- Both sensors show instability, but the preconditioned sensor exhibits a much smaller measurement deviation compared to the non-preconditioned sensor.
- Both sensors show instability, but the duration of instability is much shorter for the preconditioned sensor, particularly during rapid changes in environmental conditions.

These findings suggest that the implementation of air-preconditioning subsystems in LCPMSs deployed in smart cities can provide a cost-effective solution to overcome humidity-related inaccuracies, thereby improving the overall quality of measured air pollution data. The insights gained from the different congruence classes can inform the development of more advanced data processing-and-correction algorithms, further enhancing the performance of low-cost particulate matter sensors.

The proposed design resulted in the improved stability and accuracy of the system. When the outside air temperature is higher than the conditioned air temperature, the system provides stable and reliable measurements, despite the high relative humidity. Therefore, the paper shows that the air conditioning effectively mitigates the influence of high relative humidity, allowing the conditioned system to provide reliable data even when the unconditioned system is affected by the environmental conditions. Lastly, when the conditions are severe and both the conditioned and unconditioned systems show instability, the conditioned system’s measurements are less affected and go back sooner to the actual values.

By integrating this sensing technology into the broader infrastructure of smart cities, urban planners and policymakers can better manage air quality, enhance public health protections, and ensure that the smart-city environment remains safe and livable for all residents.

To further validate the experiment results, extended field trials should be conducted in various environments with different climate profiles. To build upon the findings of this study, future research should focus on the following areas:

- Expanding the measurement period and geographical coverage to assess the performance of preconditioned sensors in diverse climatic conditions and over longer time scales.
- Investigating the impact of other environmental factors, such as wind speed, on the performance of preconditioned sensors.
- Developing more sophisticated data-processing algorithms that can adaptively correct for sensor instability based on real-time environmental conditions.
- Exploring the potential for integrating preconditioned sensors with other smart-city technologies, such as traffic management systems and urban planning tools, to optimize air-quality monitoring and mitigation strategies.
- Conducting cost–benefit analyses to quantify the economic and societal benefits of deploying preconditioned sensors in smart cities, considering factors such as improved public health outcomes and reduced environmental impact.

## Data Availability

The data presented in this study are available on request from the corresponding authors.
